# Whole-Genome Sequencing of *Brachyspira hyodysenteriae* Isolates From England and Wales Reveals Similarities to European Isolates and Mutations Associated With Reduced Sensitivity to Antimicrobials

**DOI:** 10.3389/fmicb.2021.713233

**Published:** 2021-08-31

**Authors:** Emma Stubberfield, Jonathan Sheldon, Roderick M. Card, Manal AbuOun, Jon Rogers, Susanna Williamson, Gemma L. Kay, Mark J. Pallen, Muna F. Anjum

**Affiliations:** ^1^Department of Bacteriology, Animal and Plant Health Agency, Addlestone, United Kingdom; ^2^Animal and Plant Health Agency, Bury St Edmunds, United Kingdom; ^3^Quadram Institute Bioscience, Norwich, United Kingdom; ^4^Norwich Medical School, University of East Anglia, Norwich, United Kingdom; ^5^School of Veterinary Medicine, University of Surrey, Guildford, United Kingdom

**Keywords:** *Brachyspira hyodysenteriae*, whole genome sequencing, phylogeny, antimicrobial resistance, England and Wales

## Abstract

Brachyspira hyodysenteriae is the principal cause of swine dysentery, a disease that threatens economic productivity of pigs in many countries as it can spread readily within and between farms, and only a small number of antimicrobials are authorized for treatment of pigs. In this study, we performed whole-genome sequencing (WGS) of 81 *B. hyodysenteriae* archived at the Animal and Plant Health Agency (APHA) from diagnostic submissions and herd monitoring in England and Wales between 2004 and 2015. The resulting genome sequences were analyzed alongside 34 genomes we previously published. Multi-locus sequence typing (MLST) showed a diverse population with 32 sequence types (STs) among the 115 APHA isolates, 25 of them identified only in England; while also confirming that the dominant European clonal complexes, CC8 and CC52, were common in the United Kingdom. A core-genome SNP tree typically clustered the isolates by ST, with isolates from some STs detected only within a specific region in England, although others were more widespread, suggesting transmission between different regions. Also, some STs were more conserved in their core genome than others, despite these isolates being from different holdings, regions and years. Minimum inhibitory concentrations to commonly used antimicrobials (Tiamulin, Valnemulin, Doxycycline, Lincomycin, Tylosin, Tylvalosin) were determined for 82 of the genome-sequenced isolates; genomic analysis revealed mutations generally correlated well with the corresponding resistance phenotype. There was a major swine dysentery intervention program in 2009–2010, and antimicrobial survival curves showed a significant reduction in sensitivity to tiamulin and valnemulin in isolates collected in and after 2010, compared to earlier isolates. This correlated with a significant increase in post-2009 isolates harboring the pleuromutilin resistance gene *tva(A)*, which if present, may facilitate higher levels of resistance. The reduction in susceptibility of *Brachyspira* from diagnostic submissions to pleuromutilins, emphasizes the need for prudent treatment, control and eradication strategies.

## Introduction

Swine dysentery is characterized by mucohaemorrhagic diarrhea in pigs. It has been described in pig populations globally including Australia, Spain, Italy, and the United States (La et al., 2009, 2016a,b; Hampson and Thomson, 2012; Kou Yahui et al., 2012; Osorio et al., 2012; Mirajkar and Gebhart, 2014; Rugna et al., 2015; Hampson et al., 2016; Mahu et al., 2016; Gasparrini et al., 2017; Mahu et al., 2017; Card et al., 2018; Joerling et al., 2018), as well as being endemic to the United Kingdom (Zimmerman, 2012; APHA and SRUC, 2019). Swine dysentry is caused by the fastidious anaerobe *Brachyspira hyodysenteriae* (Harris et al., 1972; Ochiai et al., 1997), although the term swine dysentery has been used for disease caused by less prevalent *Brachyspira* species, namely *B.* hampsonii (Chander et al., 2012) and *B. suanantina* (Mushtaq et al., 2015), which can elicit similar signs and pathology. In the United Kingdom, the cost of swine dysentery per affected pig has been estimated at £4–£12 (Alderton, 2012), which includes increased feed cost due to reduced weight gain, antimicrobial treatment and/or culling of herds (Hampson and Thomson, 2012; Zimmerman, 2012; Alvarez-Ordonez et al., 2013). However, a swine dysentery targeted guidance to pig producers and allied industries to enhance awareness of this disease and increase biosecurity was promoted by the pig industry in 2009–2010 in the United Kingdom, as part of a control initiative (Waddilove, 2011).

Antimicrobials are an essential component in the treatment and control of disease due to *B. hyodysenteriae*. However, many countries report reduced susceptibility of *B. hyodysenteriae* to commonly used antimicrobials, including macrolides (Pringle et al., 2012; Kajiwara et al., 2016; Mirajkar et al., 2016; Daniel Amanda et al., 2017), and the pleuromutilins, tiamulin and valnemulin (Hidalgo et al., 2011; Mirajkar et al., 2016; Daniel Amanda et al., 2017; Card et al., 2018; Joerling et al., 2018). Development of antimicrobial resistance (AMR) clearly further restricts options available to veterinarians for treatment of swine dysentery (European Medicines Agency, 2014a,b, 2017; Card et al., 2018). Reduced susceptibility to tiamulin and valnemulin is believed to occur by acquisition of the *tva*(A) gene or through mutation at specific nucleotides in the genes for the L3 protein or the 23S rRNA or in the *fus*A gene (Card et al., 2018). Clinical resistance can result from a two-step process involving acquisition of *tva*(A) resulting in reduced susceptibility, followed by acquisition of a mutation in the genes for the L3 protein or the 23S rRNA (Card et al., 2018).

Whole-genome sequencing (WGS) has become a primary tool in the investigation of zoonotic outbreaks (Treacy et al., 2019) and in the identification of new and emergent pathogens and AMR genes in livestock (Duggett et al., 2017; Card et al., 2018; AbuOun et al., 2020). As WGS also has the potential to identify markers of AMR accurately and rapidly, and a role is being considered for WGS to replace phenotypic methods with routine AMR genotyping to help increase throughput and identify any newly emergent resistance mechanisms within archived isolates (Duggett et al., 2017; Card et al., 2018; Stubberfield et al., 2019; Bortolaia et al., 2020). For *B. hyodysenteriae* genome analysis of isolates can also assist veterinarians by providing evidence of possible epidemiological links between outbreaks, identifying probable sources of infection and distinguishing recrudescence of infection from new introductions.

In this study WGS was performed on *B. hyodysenteriae* isolates at the Animal and Plant Health Agency (APHA) reference laboratories, recovered from diagnostic submissions and herd monitoring between 2004 and 2015; this time period was selected to help monitor genomic changes over approximately 10 years. The isolates originated from fecal or intestinal samples from pigs examined for diagnostic reasons, herd monitoring purposes, or follow-up of disease outbreaks on farms, which were all from the United Kingdom, most in England and one from Wales. The aim was to compare genetic diversity in our isolates by comparing multi-locus sequence types (MLST) in APHA isolates with *B. hyodysenteriae* isolated from pig-producing countries, as well as to perform detailed phylogenetic and AMR analysis.

## Materials and Methods

### Bacterial Isolates and Culture Methods

Eighty-one *B. hyodysenteriae* isolates archived in the Bacteriological Culture Collection at APHA Bury St. Edmunds Veterinary Investigation Centre which had been recovered between 2004 and 2015 from diagnostic submissions and herd monitoring. A single submission was from Wales; all remaining submissions were from England. Diagnostic investigations of this nature into disease on a farm by a veterinary surgeon treating animals under their care is not for scientific purpose and therefore not covered by the Animal (Scientific Procedures) Act 1986. Sampling is for the immediate or long-term benefit of the individual animal, its immediate cohort or the wider epidemiological group is covered in the United Kingdom as an act of veterinary clinical practice under the Veterinary Surgeons Act, 1966, and does not require ethical permission.

*B. hyodysenteriae* recovered from APHA archives, was grown on fastidious anaerobic blood agar (FABA) plates for 3–5 days at 37–38°C under anaerobic conditions. For broth cultures, *B. hyodysenteriae* was grown in pre-reduced blood heart infusion broth with 10% horse serum under anaerobic conditions at 37–38°C overnight and shaking at 80 rpm.

Isolates were grouped into geographical regions based on Nomenclature of Territorial Units for Statistics 1 classification (Office for National Statistics, 2020) and, except for London, *B. hyodysenteriae* was present within all regions of England, with exclusion of one isolate, which originated from Wales.

### Antimicrobial Susceptibility Testing

VetMIC Brachy plates were used for MIC testing by broth dilution as described previously (Card et al., 2018). Forty-eight isolates were chosen to ensure selection of isolates from across UK regions and that a range of pleuromutilin resistance genotypes was included. The MIC results from 34 previously published UK isolates with associated genome data (Card et al., 2018) were also included in MIC analysis.

Previously established interpretative criteria (Rønne and Szancer, 1990; Pringle et al., 2007; Pringle et al., 2012) were used to categorize isolates as sensitive (at or below the proposed ECOFF value), reduced susceptibility (above the proposed ECOFF value but at or below proposed clinical breakpoint) or resistant (above the proposed clinical resistance breakpoint).

### Whole Genome Sequencing and Analysis

DNA was extracted from pelleted cells using Prepman Ultra (ThermoFisher Scientific, United Kingdom) according to manufacturer’s method. Isolates were sequenced on an Illumina MiSeq using either the 2 × 250 bp or the 2 × 300 bp protocol after Nextera XT library preparation. These whole genome sequences were analyzed alongside 34 previously published genomes from United Kingdom (UK) (Card et al., 2018). In total, 115 UK isolates were analyzed as part of this study. Sequenced isolates were submitted to the NCBI Short Read Archive (submission ID: SUB9691480; BioProject ID: PRJNA731465); Supplementary Table 1 provides details on the quality of the WGS data submitted.

Eighty-two previously published *B. hyodysenteriae* genomes published globally were downloaded from NCBI (Bellgard et al., 2009; Black et al., 2015; La et al., 2016b; Card et al., 2019); for all NCBI genomes that did not have an associated publication to provide details, the dates from pubMLST were used to approximate the isolation date. The sequences were analyzed using the Nullabor pipeline (version 1.2) (Seemann et al., 2018), using the published genome WA1 (Bellgard et al., 2009) (accession number NC_01225) as reference; genome assembly and annotation was performed using SPAdes (version 3.9.0) (Bankevich et al., 2012) and Prokka (version 1.11) (Seemann, 2014), respectively. An alignment of SNPs in the core-genome, using WA1 as reference (Accession number NC_01225), was constructed by Snippy (version 3.1) (Seemann, 2015) and SNP distance matrix made by snp-dists (version 3).^1^ The size of the core genome for *B. hyodysenteriae* isolates included in this study was 43,618 bp, and the average size of the *B. hyodysenteriae* genome for isolates sequenced in this study was 3.08 million bp (Supplementary Table 1). A phylogenetic tree was produced using the 43,618 bp core genome SNP alignment using default settings and maximum likelihood trees were bootstrapped using in RAxML-NG (version 0.9.0) (Kozlov et al., 2018). The tree was viewed and annotated in Interactive tree of life (version 5.3) (Letunic and Bork, 2016). All bioinformatics programs were used with default parameters.

The presence of AMR SNPs associated with reduced susceptibility to the antimicrobials tested were examined by extraction of 16S and 23S rRNA, *fus*(A), L2, L3, L4 genes and clustralW alignment in MegAlign (version 11, DNAStar Inc.) using *E. coli* numbering for rRNA and WA1 for protein alignments. Genomes were also screened for the presence of *lnu*C and *tva*(A) (Card et al., 2018; De Luca et al., 2018).

*In silico*, MLST was performed using SRST2 (version 0.1.5) (Inouye et al., 2014). *B. hyodysenteriae* STs were downloaded from pubMLST (*n* = 539 isolates) (accessed January 2020)^2^ (Jolley et al., 2018) and additional STs were obtained from published literature, including: 52 isolates from Spain and Portugal (Osorio et al., 2012); 180 isolates from Italy (Gasparrini et al., 2017); and 131 isolates from Germany (Joerling et al., 2018; Card et al., 2019). Isolates that share 6 alleles have been previously defined as a clonal complex (CC) (Joerling et al., 2018) and eBURST (version 3) (Feil et al., 2004) was used to identify the likely founder, as described previously (Joerling et al., 2018). Minimum spanning trees were produced using Bionumerics 6.6 (Applied Maths, 2013). Novel STs were uploaded to PubMLST.

### Statistical Analysis

After removal of duplicate isolates (same holding at same time point and genetically shown to be a clone, i.e., less than 70 SNPs apart) MIC results for 74 of the isolates were used to construct Kaplan-Meier survival curves for each antimicrobial tested. For isolates with an MIC greater than the maximum dilution, the MIC was converted to the next dilution above and isolates with MIC at the minimum dilution, were converted to the lowest dilution value of the VetMIC Brachy plate. To avoid occurrences of values below zero, MIC results were multiplied by 32, except for valnemulin MICs which was multiplied by 33 and converted into positive log2 values as done previously (Stegeman et al., 2006; Hidalgo et al., 2011). This was done using GraphPad Prism 7.03 and a log-rank test was used for statistical analysis. Isolates were split into two groups: an early group (Pre-2010) and a late group (2010–2015). This split was chosen as there was an swine dysentery control initiative promoted by the pig industry in 2009–2010 in response to the East Anglian region (Waddilove, 2011).

The correlation between MIC and AMR gene/SNP presence was calculated by two-by-two box analysis as previously described (Stubberfield et al., 2019). The test sensitivity, specificity, positive predictive value and negative predictive value were determined by: correlation between WGS-gene presence and MIC-resistant results as true positive (TP); WGS-negative and MIC-susceptible results as true negative (TN); WGS-gene present but MIC-susceptible results as false positive (FP); and WGS-negative but MIC-resistant results as false negative (FN).

## Results

In this study, 81 *B. hyodysenteriae* isolates submitted to the APHA from 2004 to 2015 underwent WGS on the Illumina platform. These were analyzed alongside 34 previously published genomes from isolates from England (Card et al., 2018), resulting in the inclusion of 115 English and Welsh *B. hyodysenteriae* genomes. Except for three isolates, the origins of all others were known: they originated from 85 pig holdings or farms; multiple isolates were collected from 15 holdings, while isolates from six holdings (A,AG,AR,CD,J, and Z) were collected over multiple years (Supplementary Table 2).

### MLST Comparison of Isolates Highlights Continental Differences and Shared STs in Europe

**FIGURE 1:**
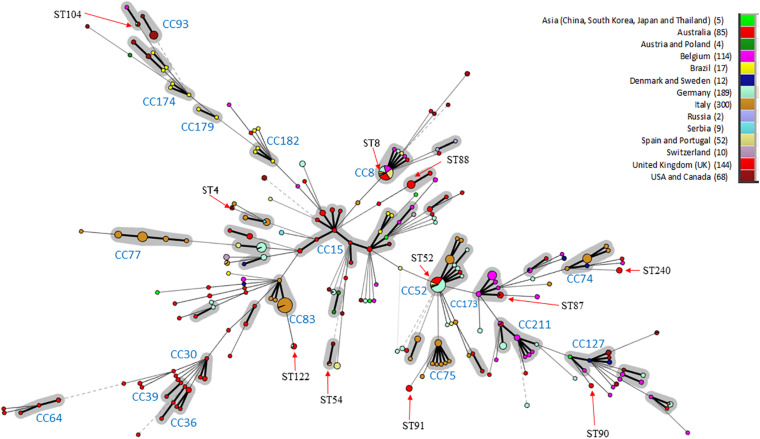
Minimum-spanning Tree of *B. hyodysenteriae* sequence types. STs have been colored according to the country where isolates originate and the number of isolates from each country is indicated in brackets. CCs are shown in gray and CC with > 3 different STs or those previously identified by *Joerling et al.* have been labeled (Joerling et al., 2018). The size of the circle corresponds to the number of isolates of that ST within the collection. STs are labeled if possessing more than two UK isolates or the ST is shared between three continents. Arrows indicate where the ST is located in the tree. Note ST for the UK includes 29 additional isolated UK STs from pubMLST.

*In silico* MLST compared the genetic diversity of the 115 English and Welsh isolates with that of 896 isolates collected globally where no genome sequence was available (Osorio et al., 2012; Gasparrini et al., 2017; Joerling et al., 2018; Card et al., 2019); it included 29 UK isolates from outside this study. Thirty-two STs were identified within the 144 UK isolates (including English and Welsh ones), and the majority (26 STs) were present in the 115 included from this study (Supplementary Table 2). A minimum-spanning tree of UK and non-UK isolates showed STs and clonal complexes (CCs) generally clustered according to continent (Figure 1). The most common STs within the UK isolates were ST52 (26 isolates) and ST8 (24 isolates); these STs, along with ST87, ST122, and ST167, have only been reported in Europe from Italy, Germany, Belgium, Serbia, Austria, Poland, Spain, United Kingdom, and Portugal, and not from North America, Asia or Australia. Twenty-five STs were unique to the UK, of which ST88 (22 isolates) and ST91 (11 isolates) were the most common. Four new STs were identified in isolates from this study, and assigned the designations ST242, ST244, ST245, and ST256.

Within Europe, some STs/CCs were observed in a single country—for example, CC74, CC75, and CC77 in Italy—whilst others were present in multiple countries e.g., ST8 and ST52. Australian isolates formed two main groups; a branch containing variants only reported in Australia (CC30, CC36, CC39, and CC64) and one (CC15) shared with other locations (e.g., Brazil and Asia). Most isolates within our dataset from United States, Canada and Brazil clustered in branches in a country-specific manner (e.g., CC182, CC179, CC174, and CC93). ST4 was identified in isolates scattered across different continents (United Kingdom, Australia and North America), as has been reported previously (Card et al., 2018). Three additional STs were common to North American and European isolates (ST54, ST55, and ST104).

### Phylogenetic Characterization of UK Isolates

**FIGURE 2:**
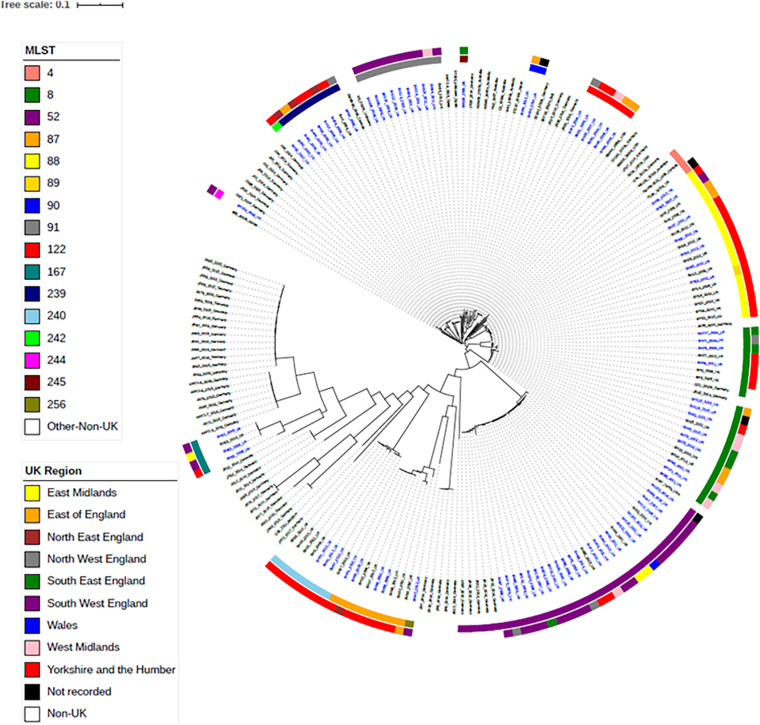
Maximum-likelihood tree of *B. hyodysenteriae* isolates based on SNP in the core-genome. One hundred and fifteen UK isolates and eighty-two non-UK isolates published on NCBI have been included (Bellgard et al., 2009; Black et al., 2015; La et al., 2016b; Card et al., 2019). Isolates have their year and country of isolation shown next to their name. Isolates sequenced as part of this study are in blue font. The rings show the UK region and the ST of isolates. Branches are colored by bootstrap values.

To investigate the relationships between the English and Welsh pig isolates from APHA with isolates from other countries, spanning Asia, America, Australia and Europe (Bellgard et al., 2009; Black et al., 2015; La et al., 2016b; Card et al., 2019), we constructed a maximum-likelihood phylogenetic tree from SNPs in the core genome (Figure 2), for isolates with WGS data available. Supplementary Table 3 provides details of the numbers of SNP differences between isolates, with respect to the reference strain WA1, in the core genome used to construct the phylogenetic tree. Isolates mainly fell into clades that reflected their STs, but as discussed below some STs harbored genetically more diverse isolates than others.

Isolates within ST4, ST91, and ST122 clustered according to their ST and were highly similar; on average there were 39, 11, and 32 core-genome SNPs between isolates within these STs, respectively (Table 1 and Supplementary Table 3). The ST91 clones (average core-genome SNPs = 11; range = 0–28 SNPs) were mostly from eight holdings (*n* = 9 isolates) in South-West England, which were known to be epidemiologically linked; they had persisted in the same region for 4 years between 2008 and 2012. Only one isolate from this ST (BH94) from 2011 was from a different UK region, which was a single holding in the Midlands (Supplementary Table 2). From bootstrap values (> 75%), there was a high level of confidence in the ST91 sub-cluster, although less confidence in position of the individual isolates within the cluster.

**TABLE 1:** Core genome SNPs within STs found in 115 UK isolates, for STs with > 2 isolates present.

ST	No. isolates	Core genome SNPs		
		
		Min	Max	Average
4	3	16	52	39
8	20	0	818	308
52	31	2	1,264	310
87	9	7	2,158	882
88/89	18	1	306	194
91	10	0	28	11
122	6	5	64	32
167	4	63	2,768	1,412
239	8	1	131	38
240	8	1	594	338

**ST90, 242, 244, 245, and 256 excluded as = 2 isolates; ST88 and ST89 were combined as they grouped closely on the tree. ST88/89 grouped together due to high similarity shown in SNPs and phylogenetic tree grouping.**

In contrast to the ST91 isolates, the ST122 cluster (average core genome SNPs = 32; range 5–64 SNPs) included isolates from five holdings and four regions of England, collected over a 5-year period. It included isolates from the Midlands in 2004, Northwest England and Yorkshire and the Humber in 2006/2007 and East of England in 2009. Confidence in this sub-cluster was provided by the high bootstrap values for the sub-cluster and the individual isolate nodes. As reported previously (Card et al., 2018), the three ST4 isolates showed significant conservation of the core genome (average core genome SNPs = 39; range 16–52 SNPs), despite originating from different continents and countries (including the United Kingdom) over a wide timespan (∼1970–2010).

Most other STs assigned to isolates included in this study, such as ST8, ST52, ST87, ST88, and ST240, had greater core-genome diversity. On average, there were ≥ 194 SNPs between isolates of these STs (Table 1 and Supplementary Table 3), although smaller groups of isolates with less diversity (≤ 70 SNPs) were observed within each ST sub-clade. For example, within the most common ST, ST52 (average = 310 SNPs; range = 2–1264 SNPs), there were 26 UK isolates from multiple holdings within five different regions, including Wales, and five German *B. hyodysenteriae* (Supplementary Tables 1, 3), which were recovered between 2006 and 2015. Although the German isolates clustered within the ST52 clade, there were more than 250 SNPs in the core genome between German and United Kingdom isolates of the same ST, indicating they were distantly related. The remaining isolates within this cluster, all from the UK, fell into six smaller sub-clades, with much lower core genome diversity between isolates within each sub-clade (≤ 70 SNPs apart). Given that these isolates were from multiple holdings and regions, it suggests that ST52 variants have been moving within different regions over this time period and the high bootstrap value of this sub-cluster provided further confidence that they retained genomic similarity within the core.

We noted that all isolates within ST240—a diverse group with a maximum of 594 SNPs in the core genome—were from Yorkshire and the Humber and had been isolated between 2008 and 2014. Within ST240 clade, there were two sub-clades, with each sub-clade comprising isolates with < 15 SNPs differences. Isolates from each sub-clade were collected from the same holding at the same time indicating a single clone had probably been responsible for outbreaks in these holdings: isolates BH30, BH31, and BH32 (holding H); and isolates BH37, BH41, and BH67 (holding G). Two isolates (BH6 and BH70) collected from different holdings and times, but from the same region, were > 280 SNPs apart, but clustered with other ST240 isolates.

The advantage of phylogenetic analysis over MLST was evident from isolate BH63 of ST89. In the seven-gene MLST scheme, ST89 differs from ST88 by a single SNP difference in the *pgm* allele. However, in the phylogenetic analysis, ST89 clustered in the ST88 cluster and showed high similarity (41–46 SNPs) to a previously described group of six ST88 isolates from Yorkshire and Humberside from holding A and one isolate from holding B (BH64) (Card et al., 2018), revealing a potential close epidemiological link that would have been missed if only considering MLST.

Within the phylogenetic tree, there was a distinct clade of weakly hemolytic isolates (Card et al., 2019; Figure 2), which included four UK isolates belonging to ST167. Within the ST167 UK isolates, there was a cluster of three isolates with < 87 SNP differences and a more diverse isolate (>2,700 SNPs different), which again showed the discriminatory capacity of phylogenetics as this diversity in the core genome would not have been apparent by MLST. The remaining weakly hemolytic isolates from Europe were from different STs and > 7,000 SNPs different in their core genomes to UK isolates.

### Reduction in Susceptibility to Antimicrobials in UK Isolates

Resistance phenotypes were determined for 48 sequenced isolates using broth dilution for tiamulin, valnemulin, doxycycline, lincomycin, tylosin, and tylvalosin to obtain the minimum inhibitory concentrations (MICs) using published breakpoints (Supplementary Table 4). These MICs were combined with published data for 34 isolates from Card et al. (2018) to obtain MICs for 82 isolates from England and Wales.

Overall, only three of the 82 isolates (3.7%) were found to be susceptible (equal to or below ECOFF values) to all six antimicrobials tested and there were 24 isolates (29.3%) that had MICs above ECOFF values for the entire antibiotic panel (Supplementary Table 4). Forty-four (53.7%) and 52 (63.4%) isolates, respectively, were above the ECOFF values for the important pleuromutilins, tiamulin, and valnemulin, with 19 (23%) of these being above the clinical breakpoint for tiamulin (Supplementary Table 5).

Several associated genetic features associated with AMR (SNPs and the *tva*(A) gene) were identified in sequenced isolates (Supplementary Table 5). For the 82 isolates tested by MIC, comparing the ECOFF values with WGS data to detect AMR gene and SNPs leading to resistance, the sensitivity and specificity values from phenotype/genotype correlations were calculated for all six antimicrobials (Supplementary Table 6). For Valnemulin, there was a perfect correlation between MIC phenotype and *tva*(A) presence, with 100% specificity and sensitivity; isolates with MIC values > 2 mg/L additionally harbored a SNP in either 23S rRNA (G2032A) or L3 (leading to the amino acid change N148S). A good correlation was observed for doxycycline, tylvalosin and lincomycin with sensitivity > 89% and specificity > 93%. For doxycycline, there was one false-negative isolate (MIC 4 mg/L without 16S G1058 SNP present) and four false-positive isolates at and just below ECOFF (MIC 0.25–0.5 mg/L). For tylvalosin, there was only one isolate that was MIC sensitive (BH119 at ECOFF 1 mg/L) but had the associated 23S rRNA A2058T SNP present. For lincomycin, there were five isolates that were just above ECOFF (MIC 2–4 mg/L) that did not have the associated gene or SNP present. For tiamulin and tylosin, there was 100% specificity but lower sensitivity (68.4 and 50%, respectively), due to a number of false positives (i.e., phenotype sensitive, but with AMR gene or SNP present). However, for the majority of these false positive isolates, the MIC was at or just below the respective ECOFF values for these antibiotics.

**FIGURE 3:**
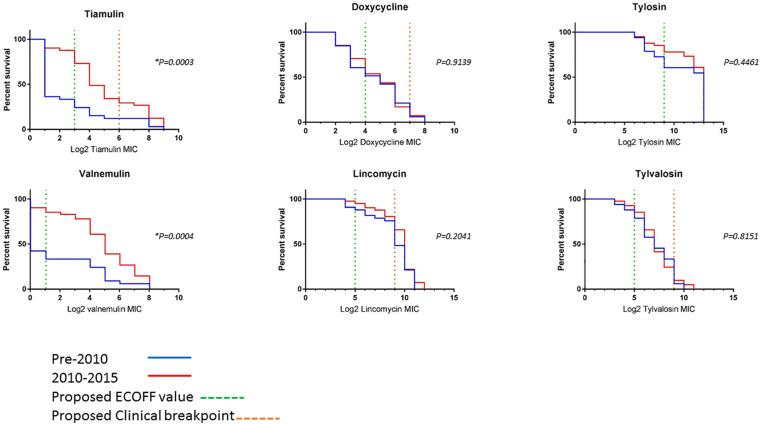
Survival curves of log2 MIC values of six antimicrobials for 73 UK isolates. The pre-2010 group is the blue line and the 2010–2015 group is the red line. The green dashed line is the proposed ECOFF value and the orange dashed line is the proposed clinical resistance breakpoints (where applicable) (Supplementary Table 6). *Indicates a statistically significant value.

To compare changes in AMR over time, survival analysis was performed using MIC data for 74 unique isolates (left after multiple isolates from the same holding/same period with less than 70 SNPs apart were removed). The isolates were divided into two categories, pre and post swine dysentery control program. The numbers of isolates in the early pre-2010 group was *n* = 33 and spanned 2005–2009 (apart from historical isolate P18A); the number in the 2010–2015 group was *n* = 41 (Figure 3). For doxycycline, lincomycin, tylosin and tylvalosin, there were no significant differences between the two groups. However, statistically significant reductions in susceptibility in isolates from 2010 to 2015 compared to 2005–2009 were seen for tiamulin (*P* = 0.0003) and valnemulin (*P* = 0.0004).

Changes in the resistance genotype over time, using the 2009 cut-off were applied for all 115 English isolates included in this study. Presence of the pleuromutilin resistance gene *tva*(A) and/or SNPs in 23S rRNA, or 50S rRNA L3 protein between the pre-2010 and 2010–2015 groups was examined. There was a very significant increase (*p* ≤ 0.0001) between these groups for tiamulin and valnemulin, with the *tva(A)* gene occasionally present pre-2009 (27.5–33.3%) but common in the 2010–2015 group (76.6–82.8%) (Table 2). The G1058C/T SNP in the 16S rRNA gene is associated with reduced susceptibility to doxycline and significantly more isolates from 2010 to 2015 harbored it than the pre-2010 group (*p* = 0.0405) (Table 2). The 23S rRNA A2058T/A2059C SNPs associated with reduced susceptibility to lincomycin and macrolides, tylosin and tylvalosin, showed no significant difference; these SNPs were found in 83.5% of the isolates (Table 2).

**TABLE 2:** AMR gene/SNP presence for 115 UK isolates split pre and post-2009 groups.

Antibiotic		Tiamulin	Valnemulin	Doxycycline	Lincomycin, tylosin, tylvalosin
Resistance mechanism	*tva(A)*/L3 N148S/G2032A (23S)	*tva(A)*/L2 T50N/G2032A (23S)	G1058C/T (16S)	A2058T/A2059C (23S)
Isolates with gene or SNP	Pre-2010 isolates *n* = 51	17 (33.3)	14 (27.5)	22 (43.1)	39 (76.5)
	2010–2015 group isolates *n* = 64	53 (82.8)	49 (76.6)	39 (60.9)	57 (89.1)
	Total *n* = 115	70 (60.9)	63 (54.8)	61 (53.0)	96 (83.5)
	*P*	<0.0001*	<0.0001*	0.0405	0.0662

*Statistically significant p-value.

## Discussion

In this study, the WGS of 81 newly sequenced English and Welsh *B. hyodysenteriae* isolates in the APHA archive collected between 2004 and 2015 were analyzed alongside 34 isolates previously sequenced (Card et al., 2018). Submissions to the APHA for *Brachyspira* culture are voluntary and swine dysentery is a non-statutory disease, and also not an OIE or EU listed disease, thus the isolate collection is not representative of all cases of *B. hyodysenteriae* in the country; however, this remains the most comprehensive *B. hyodysenteriae* collection sequenced globally. Previously published isolates focused on investigation of AMR (Card et al., 2018) and the population structure was not fully explored. By sequencing a larger proportion of APHA’s *B. hyodysenteriae* isolates, we have investigated the population structure in more detail, providing more information for outbreak investigations.

As expected, MLST showed that there was a diverse population of *B. hyodysenteriae* in the UK with 32 STs identified; some STs were reported in other countries while others were unique to the UK, and in our collection of English and Welsh isolates. The two main STs identified in UK isolates, ST8 and ST52, have been previously identified in Germany (Joerling et al., 2018), Spain (Hidalgo et al., 2011), Belgium (Mahu et al., 2017), and Italy (Rugna et al., 2015), which are EU member states with whom movement of animals are permitted. However, as only a limited numbers of *B hyodysenteriae* genomes or MLST data have been reported by pig producing countries, it is not possible to know if the 25 STs (including ST88, ST91, ST239, and ST240) only recorded in the United Kingdom or English population are unique to these regions. Nevertheless, it is clear that the STs common in isolates from the United States (ST93) and Australia (ST50, ST144, and ST150), have not been detected to date in UK isolates or elsewhere in Europe, probably reflecting the more limited intercontinental trade in live pigs (Mirajkar and Gebhart, 2014; La et al., 2016a).

Phylogenetic analysis of English and Welsh pig isolates indicated that *B. hyodysenteriae* clustered mostly by ST, showing greater core genome similarity with other isolates of the same ST than non-UK ones. Isolates from certain STs were only identified within discrete regions, while other STs appeared more widespread across England and Wales, suggesting transmission of isolates between different regions. Furthermore, we noted greater stability of the core genome in isolates from particular STs. For example, ST91, ST122, and ST239 isolates had the least core genome variation and were the most conserved, despite isolates being collected over several years and from multiple holdings, with ST122 and ST239 isolates also being from different regions. In contrast, the core genome of isolates from STs such as ST88/89 and ST240, which had also been collected over a number of years but mostly from one region, were much more diverse. Although reasons for differences in stability of the core genome and wider geographical distribution of certain STs are unknown, they may reflect greater dissemination from some farms, differences in biosecurity measures and/or awareness of swine dysentery in different pig keeping sectors, and different responses to swine dysentery diagnosis. Eliminating infection through partially or fully depopulating may be more achievable for commercial herds than smaller or non-commercial herds, including those with pigs of special breed status (Smith et al., 2013).

Antimicrobials of the pleuromutilin class, tiamulin or valnemulin, are often selected by veterinarians to treat swine dysentery. Isolates with reduced sensitivity to tiamulin and valnemulin have been described (Card et al., 2018). Analysis of this larger dataset shows a shift in pleuromutilin susceptibility phenotype, with isolates from 2010 to 2015 showing reduced susceptibility, compared to 2004–2009 isolates, predominantly due to the increased presence of the *tva*(A) gene, which can lead to acquisition of further changes resulting in a higher level of resistance to pleuromutilins (Card et al., 2018). The increased presence of the *tva*(A) gene post 2009 may predispose isolates to development of resistance. A similar shift in pleuromutilins MIC, tiamulin, and valnemulin, has been seen in survival curves of *B. hyodysenteriae* isolates from Spain, Germany, Czech Republic, and Italy (Hidalgo et al., 2011; Prášek et al., 2014; Rugna et al., 2015; Joerling et al., 2018). Although reduced sensitivity has been widely reported for macrolides and lincomycin (Hidalgo et al., 2011; Rugna et al., 2015; Kajiwara et al., 2016; Mirajkar et al., 2016; Daniel Amanda et al., 2017; Card et al., 2018), in our study there was no significant change in the phenotype or underlying genotype for either over the time period of this study. For doxycycline, even though there was no significant change seen in the MIC over the time period of the study, the number of isolates harboring the 16S rRNA G1058C/T SNP increased. We speculate that if a larger panel of isolates had been included, an increase may have been observed in the MIC values.

For the isolates in this study the resistance genotype correlated well with MIC phenotype for four of the six antibiotics tested (>89% sensitivity and >93% specificity). Tiamulin and tylosin showed lower sensitivity due to a number of false positive isolates (those fully sensitive by MIC but containing the resistant gene/SNP associated with the resistance). However, many of the outliers were at or just below the ECOFF value, similar to findings from *Escherichia coli* (Davies et al., 2020). In future, as more sequencing and MIC data becomes available, the ECOFF values may need reviewing and new cut-offs suggested, and we may also be able to better correlate other mutational changes that occur in WGS data to help improve correlations with phenotypes. Another aspect of work for future consideration would be identification of virulence factors that enable disease. In pathogens, they can be present on mobile genetic elements, leading to genetic diversity, with some isolates more closely linked to disease (Wu et al., 2008; Wragg et al., 2009; Figueiredo et al., 2015; Card et al., 2016).

## Conclusion

In conclusion, in this study we sequenced *B. hyodysenteriae* isolates in the APHA’s culture collection from pigs in England and Wales, from between 2004 and 2015. This allowed an in-depth analysis of the *B. hyodysenteriae* population, and provided WGS data for future research and outbreak investigations, which may help establish epidemiological links between outbreaks and sources of infection. Swine dysentery continues to present a potential threat to the UK pig industry, with a period of increased diagnoses recorded during 2017–2019 which subsided in 2020 (APHA and SRUC, 2019). Whole genome sequencing provides a means to better understand the epidemiology of the disease and detect changes in antimicrobial resistance in *B. hyodysenteriae*, which are both vital to controlling this economically important disease. It is noteworthy that the UK pig sector has made excellent progress in the past few years to reduce the use of antimicrobials (UK-VARSS, 2019), so an expected benefit would be increased susceptibility of *B. hyodysenteriae* to some of the key antimicrobials used for therapeutics. If WGS were applied to *B. hyodysenteriae* collected from other major pig producing countries, then similarly informative datasets would also be available for both genetic diversity and underlying mechanisms for resistance present in the *B. hyodysenteriae* population.

## Data Availability Statement

The datasets presented in this study can be found in online repositories. The names of the repository/repositories and accession number(s) can be found below: NCBI with accession PRJNA731465 (https://www.ncbi.nlm.nih.gov/bioproject/PRJNA731465/).

## Author Contributions

ES and JS: laboratory work, sequencing of isolates, genomic analysis. JR: strain isolation. MA, RC, MFA, MP, and GK: supervision and guidance of genomic data. SW and RC: brachyspira specialism. MFA and MP: concept and funding. All authors contributed to writing of the manuscript. All authors contributed to the article and approved the submitted version.

## Conflict of Interest

The authors declare that the research was conducted in the absence of any commercial or financial relationships that could be construed as a potential conflict of interest.

## Publisher’s Note

All claims expressed in this article are solely those of the authors and do not necessarily represent those of their affiliated organizations, or those of the publisher, the editors and the reviewers. Any product that may be evaluated in this article, or claim that may be made by its manufacturer, is not guaranteed or endorsed by the publisher.

## Footnotes

1
https://github.com/tseemann/snp-dists

2
https://pubmlst.org/brachyspira/

## Supplementary Material

Supplementary Table 1 Quality of whole genome sequences of Brachyspira hyodysenteriae isolates sequenced in this study.

Supplementary Table 2 Epidemiological details of UK isolates including: date of isolation, UK region, holding and MLST ST. Note BH2 to BH38 are from Card et al. (2018) and P18A was isolated by Lemcke and Burrows (1981). Isolates arranged according to ST.

Supplementary Table 3 Core genome SNP distances associated with the phylogenetic tree (Figure 2). Isolates arranged by sequence type.

Supplementary Table 4 MIC proposed ECOFF values and clinical breakpoints used in this study.

Supplementary Table 5 MICs obtained by broth dilution for tiamulin, valnemulin, doxycycline, tylvalosin, lincomycin, and tylosin; and AMR gene/SNPs associated with reduced susceptibility for the 82 isolates sequenced in this study. Isolates have been ordered based on their tiamulin MIC. Values higher than the ECOFF value have been shown in bold. *rpl*B and *rpl*C refers to SNPs in L2 and L3 proteins, respectively, associated with tiamulin reduced susceptibility; 16S rRNA refers to G1058 SNP associated with doxycycline reduced susceptibility; 23S rRNA refers to SNPs associated with reduced susceptibility to macrolide and lincomycin; and *tva*(A) refers to the gene associated with reduced susceptibility to pleuromutilins. *NT* = isolates not tested by MIC (but AMR genotype is shown). ^∗^ = isolates MIC used in survival analysis.

Supplementary Table 6 Statistical analysis using two-by-two tables of WGS and ECOFF values for 82 isolates. PPV, positive predictive value; NPV, negative predictive value.
