# Supplementary material for: Whole-Genome Sequencing of Brachyspira hyodysenteriae Isolates From England and Wales Reveals Similarities to European Isolates and Mutations Associated With Reduced Sensitivity to Antimicrobials
Source: Front Microbiol. 2021 Aug 31;12:713233. doi: 10.3389/fmicb.2021.713233 (PMC8439570; doi:10.3389/fmicb.2021.713233)
Supplement: Supplementary Table 1 — Quality of whole genome sequences of Brachyspira hyodysenteriae isolates sequenced in this study. [file Data_Sheet_1.zip › Table 2.DOCX]

**Table S2.** Epidemiological details of UK isolates including: date of isolation, UK region, holding and MLST ST. Note BH2 to BH38 are from *Card et al 2018* (18) and P18A was isolated by *R.M Lemcke* and *M.R.Burrows* (60). Isolates arranged according to ST.

| **Isolate** | **Year-Month** | **Region** | **Holding** | **Sequence Type** | **Reference** |
| --- | --- | --- | --- | --- | --- |
| P18A | 1970s | Unknown | Unknown | 4 | Card et al |
| BH62 | 2011-5 | Yorkshire and the Humber | AA | 8 | This study |
| BH25 | 2011-6 | West Midlands | AB | 8 | Card et al |
| BH117 | 2004-4 | South East England | AC | 8 | This study |
| BH121 | 2011-1 | South East England | AD | 8 | This study |
| BH93 | 2011-5 | West Midlands | AF | 8 | This study |
| BH95 | 2011-6 | West Midlands | AG | 8 | This study |
| BH45 | 2010-6 | West Midlands | AH | 8 | This study |
| BH76 | 2009-7 | South East England | AI | 8 | This study |
| BH71 | 2004-5 | North West England | AJ | 8 | This study |
| BH27 | 2012-1 | Yorkshire and the Humber | CO | 8 | Card et al |
| BH34 | 2012-9 | East of England | CQ | 8 | Card et al |
| BH123 | 2015-9 | East of England | KK | 8 | This study |
| BH78 | 2012-10 | South East England | PP | 8 | This study |
| BH79 | 2012-11 | South East England | PP | 8 | This study |
| BH15 | 2010-5 | East of England | X | 8 | Card et al |
| BH49 | 2011-10 | Yorkshire and the Humber | Y | 8 | This study |
| BH2 | 2005-11 | Yorkshire and the Humber | Z | 8 | Card et al |
| BH3 | 2005-11 | Yorkshire and the Humber | Z | 8 | Card et al |
| BH124 | 2015-9 | Unknown | Unknown | 8 | This study |
| BH108 | 2008-9 | South West England | AS | 52 | This study |
| BH120 | 2007-2 | South East England | AY | 52 | This study |
| BH90 | 2006-6 | South West England | BG | 52 | This study |
| BH119 | 2006-10 | South East England | BK | 52 | This study |
| BH73 | 2006-10 | North West England | BL | 52 | This study |
| BH75 | 2007-7 | North West England | BN | 52 | This study |
| BH47 | 2007-8 | South West England | BO | 52 | This study |
| BH110 | 2008-12 | South West England | BS | 52 | This study |
| BH111 | 2008-12 | South West England | BT | 52 | This study |
| BH122 | 2009-4 | Wales | BU | 52 | This study |
| BH97 | 2009-6 | South West England | BV | 52 | This study |
| BH98 | 2009-9 | South West England | BX | 52 | This study |
| BH77 | 2010-1 | West Midlands | BY | 52 | This study |
| BH99 | 2010-2 | South West England | BZ | 52 | This study |
| BH100 | 2010-6 | South West England | CA | 52 | This study |
| BH20 | 2010-10 | South West England | CB | 52 | Card et al |
| BH101 | 2011-10 | South West England | CC | 52 | This study |
| BH39 | 2011-12 | South West England | CD | 52 | This study |
| BH42 | 2013-10 | South West England | CD | 52 | This study |
| BH24 | 2011-1 | East Midlands | CM | 52 | Card et al |
| BH80 | 2011-1 | East Midlands | CM | 52 | This study |
| BH38 | 2013-9 | Yorkshire and the Humber | CP | 52 | Card et al |
| BH68 | 2013-9 | Yorkshire and the Humber | CP | 52 | This study |
| BH103 | 2015-1 | South West England | NN | 52 | This study |
| BH116 | 2012-8 | South West England | OO | 52 | This study |
| BH102 | 2014-2 | South West England | RR | 52 | This study |
| BH16 | 2010-6 | East of England | CN | 87 | Card et al |
| BH60 | 2009-12 | North East England | I | 87 | This study |
| BH12 | 2009-7 | Yorkshire and the Humber | J | 87 | Card et al |
| BH17 | 2010-7 | Yorkshire and the Humber | J | 87 | Card et al |
| BH61 | 2010-7 | Yorkshire and the Humber | J | 87 | This study |
| BH33 | 2012-6 | Yorkshire and the Humber | K | 87 | Card et al |
| BH58 | 2009-3 | Yorkshire and the Humber | M | 87 | This study |
| BH48 | 2010-11 | Yorkshire and the Humber | N | 87 | This study |
| BH36 | 2013-1 | Yorkshire and the Humber | O | 87 | Card et al |
| BH13 | 2009-8 | Yorkshire and the Humber | A | 88 | Card et al |
| BH14 | 2009-10 | Yorkshire and the Humber | A | 88 | Card et al |
| BH18 | 2010-10 | Yorkshire and the Humber | A | 88 | Card et al |
| BH19 | 2010-10 | Yorkshire and the Humber | A | 88 | Card et al |
| BH21 | 2010-11 | Yorkshire and the Humber | A | 88 | Card et al |
| BH22 | 2010-11 | Yorkshire and the Humber | A | 88 | Card et al |
| BH26 | 2012-1 | Yorkshire and the Humber | B | 88 | Card et al |
| BH28 | 2012-2 | Yorkshire and the Humber | B | 88 | Card et al |
| BH29 | 2012-3 | Yorkshire and the Humber | B | 88 | Card et al |
| BH64 | 2012-3 | Yorkshire and the Humber | B | 88 | This study |
| BH66 | 2012-3 | Yorkshire and the Humber | B | 88 | This study |
| BH65 | 2012-3 | Yorkshire and the Humber | B | 88 | This study |
| BH9 | 2008-11 | East of England | C1 | 88 | Card et al |
| BH8 | 2008-10 | East of England | C2 | 88 | Card et al |
| BH96 | 2005-11 | South West England | D | 88 | This study |
| BH69 | 2013-12 | Yorkshire and the Humber | LL | 88 | This study |
| BH87 | 2011-10 | Yorkshire and the Humber | Z | 88 | This study |
| BH63 | 2012-2 | Yorkshire and the Humber | E | 89 | This study |
| BH84 | 2011-7 | East of England | CF | 90 | This study |
| BH86 | 2014-3 | Unknown | Unknown | 90 | This study |
| BH107 | 2008-9 | South West England | AK | 91 | This study |
| BH109 | 2008-10 | South West England | AM | 91 | This study |
| BH112 | 2009-2 | South West England | AN | 91 | This study |
| BH113 | 2010-4 | South West England | AO | 91 | This study |
| BH114 | 2011-7 | South West England | AP | 91 | This study |
| BH115 | 2011-7 | South West England | AQ | 91 | This study |
| BH89 | 2011-7 | South West England | AR | 91 | This study |
| BH35 | 2012-1 | South West England | AR | 91 | Card et al |
| BH106 | 2008-10 | South West England | AS | 91 | This study |
| BH94 | 2011-6 | West Midlands | AT | 91 | This study |
| BH91 | 2004-7 | West Midlands | AG | 122 | This study |
| BH72 | 2006-2 | North West England | AW | 122 | This study |
| BH51 | 2006-10 | Yorkshire and the Humber | AX | 122 | This study |
| BH53 | 2007-8 | Yorkshire and the Humber | AZ | 122 | This study |
| BH40 | 2009-2 | East of England | BA | 122 | This study |
| BH46 | 2009-2 | East of England | BA | 122 | This study |
| BH55 | 2008-10 | Yorkshire and the Humber | BC | 167 | This study |
| BH82 | 2008-10 | South West England | BD | 167 | This study |
| BH83 | 2008-11 | South West England | BD | 167 | This study |
| BH23 | 2010-12 | East Midlands | BF | 167 | Card et al |
| BH81 | 2008-2 | East of England | Q | 239 | This study |
| BH7 | 2008-9 | Yorkshire and the Humber | R1 | 239 | Card et al |
| BH50 | 2005-8 | North East England | P | 239 | This study |
| BH54 | 2008-9 | North West England | R2 | 239 | This study |
| BH56 | 2008-10 | Yorkshire and the Humber | T | 239 | This study |
| BH57 | 2008-12 | Yorkshire and the Humber | U | 239 | This study |
| BH59 | 2009-5 | North East England | V | 239 | This study |
| BH44 | 2009-5 | North West England | W | 239 | This study |
| BH37 | 2013-5 | Yorkshire and the Humber | G | 240 | Card et al |
| BH41 | 2013-5 | Yorkshire and the Humber | G | 240 | This study |
| BH67 | 2013-5 | Yorkshire and the Humber | G | 240 | This study |
| BH30 | 2012-3 | Yorkshire and the Humber | H | 240 | Card et al |
| BH31 | 2012-3 | Yorkshire and the Humber | H | 240 | Card et al |
| BH32 | 2012-3 | Yorkshire and the Humber | H | 240 | Card et al |
| BH6 | 2008-7 | Yorkshire and the Humber | II | 240 | Card et al |
| BH70 | 2014-9 | Yorkshire and the Humber | MM | 240 | This study |
| BH52 | 2007-4 | Yorkshire and the Humber | CI | 242 | This study |
| BH104 | 2005-11 | South West England | CJ | 244 | This study |
| BH118 | 2005-7 | South East England | CL | 245 | This study |
| BH43 | 2012-1 | South West England | CE | 256 | This study |
