# Supplementary material for: Whole-Genome Sequencing of Brachyspira hyodysenteriae Isolates From England and Wales Reveals Similarities to European Isolates and Mutations Associated With Reduced Sensitivity to Antimicrobials
Source: Front Microbiol. 2021 Aug 31;12:713233. doi: 10.3389/fmicb.2021.713233 (PMC8439570; doi:10.3389/fmicb.2021.713233)
Supplement: Supplementary Table 1 — Quality of whole genome sequences of Brachyspira hyodysenteriae isolates sequenced in this study. [file Data_Sheet_1.zip › Table 4.DOCX]

| Antimicrobial | ECOFF value*  MIC (mg/L) | Clinical resistant breakpoint*  MIC (mg/L) |
| --- | --- | --- |
| Tiamulin | >0.25 (1) | >2 (1) |
| Valnemulin | >0.125 (1) | N/A |
| Doxycycline | >0.5 (1) | >4 (2) |
| Tylvalosin | >1 (1) | >16 (3) |
| Lincomycin | > 1 (1) | >16 (3) |
| Tylosin | > 16 (1) | >16 (3, 4) |

**Table S4.** MIC ECOFF values and clinical breakpoints used in this study.

These values were gathered from the following publications:

1. Pringle M, Landen A, Unnerstad HE, Molander B, Bengtsson B. Antimicrobial susceptibility of porcine Brachyspira hyodysenteriae and Brachyspira pilosicoli isolated in Sweden between 1990 and 2010. Acta Vet Scand. 2012;54:54.

2. Pringle M, Fellstrom C, Johansson KE. Decreased susceptibility to doxycycline associated with a 16S rRNA gene mutation in Brachyspira hyodysenteriae. Vet Microbiol. 2007;123(1-3):245-8.

3. Rønne H, Szancer J. In vitro susceptibility of Danish field isolates of Treponema hyodysenteriae to chemotherapeutics in swine dysentery (SD) therapy. Interpretation of MIC results based on the pharmacokinetic properties of the antibacterial agents. In: Proceedings, International Pig Veterinary Society, 11th Congress, July 1-5, 1990, Lausanne, Switzerland Swiss Association of Swine Medicine, Berne, Switzerland. 1990.

4. Swedres-Svarm. Consumption of antibiotics and occurrence of resistance in Sweden Solna/Uppsala2016 [Available from: <http://www.sva.se/globalassets/redesign2011/pdf/om_sva/publikationer/swedres_svarm2016.pdf>.
