# Supplementary material for: Whole-Genome Sequencing of Brachyspira hyodysenteriae Isolates From England and Wales Reveals Similarities to European Isolates and Mutations Associated With Reduced Sensitivity to Antimicrobials
Source: Front Microbiol. 2021 Aug 31;12:713233. doi: 10.3389/fmicb.2021.713233 (PMC8439570; doi:10.3389/fmicb.2021.713233)
Supplement: Supplementary Table 1 — Quality of whole genome sequences of Brachyspira hyodysenteriae isolates sequenced in this study. [file Data_Sheet_1.zip › Table 5.DOCX]

**Table S5.** MICs obtained by broth dilution for tiamulin, valnemulin, doxycycline, tylvalosin, lincomycin and tylosin; and AMR gene/SNPs associated with reduced susceptibility for the 82 isolates sequenced in this study. Isolates have been ordered based on their tiamulin MIC. Values higher than the ECOFF value have been shown in bold. Note: *rpl*B and *rpl*C refers to SNPs in L2 and L3 proteins respectively, associated with tiamulin reduced susceptibility; 16S rRNA refers to G1058 SNP associated with doxycycline reduced susceptibility; 23S rRNA refers to SNPs associated with reduced susceptibility to macrolide and lincomycin; and *tva*(A) refers to the gene associated with reduced susceptibility to pleuromutilins. NT = isolates not tested by MIC (but AMR genotype is shown). *=isolates MIC used in survival analysis.

| Isolate | Phenotype | | Genotype | | | Phenotype | | | Genotype | Phenotype | Genotype |
| --- | --- | --- | --- | --- | --- | --- | --- | --- | --- | --- | --- |
|  | Tiamulin (mg/L) | Valnemulin (mg/L) | *rpl*B | *rpl*C | *tva*(A) | Tylvalosin (mg/L) | Lincomycin (mg/L) | Tylosin (mg/L) | 23S rRNA | Doxycycline (mg/L) | 16S rRNA |
| BH40 | ≤0.063 | 0.063 |  |  |  | **8** | **16** | 8 | A2058T | **1** | G1058C |
| BH43 | ≤0.063 | 0.063 |  |  |  | **16** | **32** | 16 | A2058T | ≤0.125 |  |
| BH44 | ≤0.063 | 0.063 |  |  |  | **32** | **64** | 16 | A2058T | ≤0.125 |  |
| BH46 | ≤0.063 | ≤0.031 |  |  |  | **16** | **32** | **>128** | A2058T | **2** | G1058C |
| BH50 | ≤0.063 | ≤0.031 |  |  |  | **8** | **16** | **>128** | A2058T | 0.25 |  |
| BH51 | ≤0.063 | ≤0.031 |  |  |  | **16** | **16** | **>128** | A2058T | **1** | G1058C |
| BH52 | ≤0.063 | ≤0.031 |  |  |  | **16** | **64** | 8 | A2058T | **4** | G1058C |
| BH53 | ≤0.063 | ≤0.031 |  |  |  | **8** | **32** | 8 | A2058T | **2** | G1058C |
| BH54 | ≤0.063 | ≤0.031 |  |  |  | 0.5 | ≤0.5 | 4 |  | 0.5 |  |
| BH56 | ≤0.063 | 0.063 |  |  |  | **16** | **32** | 16 | A2058T | 0.25 |  |
| BH57 | ≤0.063 | 0.063 |  |  |  | **16** | **32** | 16 | A2058T | 0.5 |  |
| BH59 | ≤0.063 | ≤0.031 |  |  |  | **32** | **64** | 16 | A2058T | 0.5 |  |
| BH64 | ≤0.063 | **0.25** |  |  | tva(A) | **2** | **32** | **128** | A2058T | **2** | G1058C |
| BH66 | ≤0.063 | ≤0.031 |  | N148S |  | **16** | **32** | **8** | A2058T | 0.25 | G1058C |
| BH73 | ≤0.063 | ≤0.031 |  |  |  | **16** | **16** | **>128** | A2058T | ≤0.125 |  |
| BH90 | ≤0.063 | ≤0.031 |  |  |  | **2** | **16** | **>128** | A2058T | ≤0.125 |  |
| BH91 | ≤0.063 | ≤0.031 |  |  |  | **16** | **32** | **>128** | A2058T | **2** | G1058C |
| BH96 | ≤0.063 | ≤0.031 |  |  |  | **2** | **16** | 4 | A2058T | **1** | G1058C |
| BH106 | ≤0.063 | ≤0.031 |  |  |  | ≤0.25 | ≤0.5 | ≤2 |  | 0.25 |  |
| BH117 | ≤0.063 | ≤0.031 |  |  |  | ≤0.25 | ≤0.5 | ≤2 |  | ≤0.125 |  |
| BH119 | ≤0.063 | ≤0.031 |  |  |  | 1 | **4** | **>128** | A2058T | 0.25 |  |
| BH48 | 0.125 | 0.063 |  | N148S |  | **2** | **32** | **>128** | A2058T | **2** | G1058C |
| BH42 | 0.25 | **0.5** |  |  | tva(A) | ≤0.25 | 1 | ≤2 |  | ≤0.125 |  |
| BH45 | 0.25 | **0.5** |  |  | tva(A) | **2** | **8** | **64** | A2058T | 0.5 | G1058C |
| BH55 | 0.25 | **0.5** | T50N |  | tva(A) | 1 | 1 | 4 |  | **4** |  |
| BH63 | 0.25 | **0.5** |  |  | tva(A) | **2** | **32** | **128** | A2058T | **2** | G1058C |
| BH65 | 0.25 | **0.25** |  |  | tva(A) | **2** | **32** | **64** | A2058T | **1** | G1058C |
| BH100 | **0.5** | **1** |  |  | tva(A) | **4** | **16** | **>128** | A2058T | ≤0.125 |  |
| BH123 | **0.5** | **1** |  |  | tva(A) | **4** | **32** | **>128** | A2058T | **1** | G1058C |
| BH124 | **0.5** | **1** |  |  | tva(A) | **4** | **32** | **>128** | A2058T | **2** | G1058C |
| BH47 | **0.5** | **2** |  |  | tva(A) | **2** | **16** | **>128** | A2058T | ≤0.125 |  |
| BH62 | **0.5** | **1** |  |  | tva(A) | **2** | **32** | **128** | A2058T | **2** | G1058C |
| BH78 | **0.5** | **1** |  |  | tva(A) | **8** | **32** | **>128** | A2058T | **2** | G1058C |
| BH80 | **0.5** | **0.5** |  |  | tva(A) | 0.5 | **2** | 4 |  | 0.5 |  |
| BH98 | **0.5** | **1** |  |  | tva(A) | **2** | **8** | **128** | A2058T | 0.5 |  |
| BH101 | **1** | **1** |  |  | tva(A) | **2** | **16** | **128** | A2058T | ≤0.125 |  |
| BH102 | **1** | **1** |  |  | tva(A) | **8** | **16** | **>128** | A2058T | **2** | G1058C |
| BH103 | **1** | **4** |  |  | tva(A) | **32** | **16** | **>128** | A2059G | ≤0.125 |  |
| BH41 | **1** | **1** |  |  | tva(A) | **4** | **32** | **>128** | A2058T | **2** | G1058T |
| BH49 | **1** | **2** |  |  | tva(A) | **4** | **32** | **128** | A2058T | **2** | G1058C |
| BH67 | **1** | **1** |  |  | tva(A) | **2** | **32** | **128** | A2058T | **4** | G1058T |
| BH79 | **1** | **2** |  |  | tva(A) | **8** | **32** | **>128** | A2058T | **4** | G1058C |
| BH68 | **2** | **2** |  |  | tva(A) | **8** | **32** | **>128** | A2058T | 0.25 |  |
| BH60 | **8** | **>4** |  | N148S | tva(A) | **2** | **32** | **128** | A2058T | **2** | G1058C |
| BH61 | **8** | **4** |  | N148S | tva(A) | **2** | **32** | **>128** | A2058T | **1** | G1058C |
| BH70 | **8** | **4** |  |  | tva(A) | **8** | **64** | **>128** | A2058T | **2** | G1058T |
| BH82 | **8** | **1** | T50N |  | tva(A) | 0.5 | **2** | 4 |  | 0.25 |  |
| BH83 | **8** | **1** | T50N |  | tva(A) | 1 | **2** | 4 |  | 0.25 |  |
| BH39 | NT | NT |  |  | tva(A) | NT | NT | NT |  | NT |  |
| BH58 | NT | NT |  | N148S |  | NT | NT | NT | A2058T | NT | G1058C |
| BH69 | NT | NT |  |  | tva(A) | NT | NT | NT | A2058T | NT | G1058C |
| BH71 | NT | NT |  |  |  | NT | NT | NT | A2058T | NT | G1058C |
| BH72 | NT | NT |  |  |  | NT | NT | NT | A2058T | NT | G1058C |
| BH75 | NT | NT |  |  | tva(A) | NT | NT | NT | A2058T | NT |  |
| BH76 | NT | NT |  | N148S |  | NT | NT | NT | A2058T | NT | G1058C |
| BH77 | NT | NT |  |  | tva(A) | NT | NT | NT | A2058T | NT |  |
| BH81 | NT | NT |  |  |  | NT | NT | NT | A2058T | NT |  |
| BH84 | NT | NT |  |  |  | NT | NT | NT | A2058T | NT | G1058C |
| BH86 | NT | NT |  |  |  | NT | NT | NT | A2058T | NT | G1058C |
| BH87 | NT | NT |  |  |  | NT | NT | NT | A2058T | NT | G1058C |
| BH89 | NT | NT |  |  |  | NT | NT | NT | A2058T | NT |  |
| BH93 | NT | NT |  |  | tva(A) | NT | NT | NT | A2058T | NT | G1058C |
| BH94 | NT | NT |  |  |  | NT | NT | NT | A2058T | NT |  |
| BH95 | NT | NT |  | N148S | tva(A) | NT | NT | NT | A2058T | NT | G1058C |
| BH97 | NT | NT |  | N148S |  | NT | NT | NT |  | NT |  |
| BH99 | NT | NT |  |  | tva(A) | NT | NT | NT | A2058T | NT | G1058C |
| BH104 | NT | NT |  |  |  | NT | NT | NT |  | NT |  |
| BH107 | NT | NT |  |  |  | NT | NT | NT |  | NT | G1058C |
| BH108 | NT | NT |  |  |  | NT | NT | NT | A2059G | NT |  |
| BH109 | NT | NT |  |  |  | NT | NT | NT |  | NT |  |
| BH110 | NT | NT |  |  |  | NT | NT | NT |  | NT |  |
| BH111 | NT | NT |  |  | tva(A) | NT | NT | NT | A2058T | NT |  |
| BH112 | NT | NT |  |  |  | NT | NT | NT | A2058T | NT |  |
| BH113 | NT | NT |  |  |  | NT | NT | NT | A2058T | NT |  |
| BH114 | NT | NT |  |  |  | NT | NT | NT | A2058T | NT |  |
| BH115 | NT | NT |  |  |  | NT | NT | NT | A2058T | NT |  |
| BH116 | NT | NT |  |  | tva(A) | NT | NT | NT |  | NT |  |
| BH118 | NT | NT |  |  |  | NT | NT | NT | A2058T | NT |  |
| BH120 | NT | NT |  |  |  | NT | NT | NT | A2058T | NT |  |
| BH121 | NT | NT |  |  | tva(A) | NT | NT | NT | A2058T | NT | G1058C |
| BH122 | NT | NT |  |  | tva(A) | NT | NT | NT |  | NT |  |
