# Supplementary material for: Whole-Genome Sequencing of Brachyspira hyodysenteriae Isolates From England and Wales Reveals Similarities to European Isolates and Mutations Associated With Reduced Sensitivity to Antimicrobials
Source: Front Microbiol. 2021 Aug 31;12:713233. doi: 10.3389/fmicb.2021.713233 (PMC8439570; doi:10.3389/fmicb.2021.713233)
Supplement: Supplementary Table 1 — Quality of whole genome sequences of Brachyspira hyodysenteriae isolates sequenced in this study. [file Data_Sheet_1.zip › Table 6.DOCX]

|  | tiamulin | valnemulin | doxycycline | tylvalosin | lincomycin | tylosin |
| --- | --- | --- | --- | --- | --- | --- |
| True positive | 44 | 52 | 44 | 70 | 71 | 60 |
| True negative | 26 | 30 | 33 | 11 | 6 | 11 |
| False positive | 12 | 0 | 4 | 1 | 0 | 11 |
| False negative | 0 | 0 | 1 | 0 | 5 | 0 |
| Sensitivity | 68.4% | 100.0% | 89.2% | 91.7% | 100.0% | 50.0% |
| Specificity | 100.0% | 100.0% | 97.8% | 100.0% | 93.4% | 100.0% |
| Ppv | 78.6% | 100.0% | 91.7% | 98.6% | 100.0% | 84.5% |
| npv | 100.0% | 100.0% | 97.1% | 100.0% | 54.5% | 100.0% |

**Table S6.** Statistical analysis using two-by-two tables of WGS and ECOFF values for 82 isolates. Abbreviations: PPV = positive predictive value, NPV = negative predictive value.
